# Quantifying the direct public health care cost of systemic sclerosis: A comprehensive data linkage study

**DOI:** 10.1097/MD.0000000000008503

**Published:** 2017-12-01

**Authors:** Kathleen Morrisroe, Wendy Stevens, Joanne Sahhar, Gene-Siew Ngian, Candice Rabusa, Nava Ferdowsi, Catherine Hill, Susanna Proudman, Mandana Nikpour

**Affiliations:** a Department of Medicine, The University of Melbourne at St Vincent's Hospital (Melbourne); b Department of Rheumatology, St Vincent's Hospital (Melbourne); c Department of Medicine, Monash University, Clayton and Monash Health, Victoria; d Rheumatology Unit, Royal Adelaide Hospital, North Terrace; e Rheumatology Unit, The Queen Elizabeth Hospital, Woodville Road, Woodville; f Discipline of Medicine, University of Adelaide, SA, Australia.

**Keywords:** financial burden, healthcare utilization, scleroderma, systemic sclerosis

## Abstract

To quantify the direct healthcare cost of systemic sclerosis (SSc) and identify its determinants. Healthcare use was captured through data linkage, wherein clinical and medication data for SSc patients from the state of Victoria enrolled in the Australian Scleroderma Cohort Study were linked with the Victorian hospital admissions and emergency presentations data sets, and the Medicare Benefits Schedule which contains all government subsidized ambulatory care services, for the period 2011-2015. Medication cost was determined from the Pharmaceutical Benefits Scheme. Costs were extrapolated to all Australian SSc patients based on SSc prevalence of 21.1 per 100,000 and an Australian population of 24,304,682 in 2015. Determinants of healthcare cost were estimated using logistic regression. Total healthcare utilization cost to the Australian government extrapolated to all Australian SSc patients from 2011 to 2015 was Australian Dollar (AUD)$297,663,404.77, which is an average annual cost of AUD$59,532,680.95 (US Dollar [USD]$43,816,040.08) and annual cost per patient of AUD$11,607.07 (USD$8,542.80). Hospital costs, including inpatient hospitalization and emergency department presentations, accounted for the majority of these costs (44.4% of total), followed by medication cost (31.2%) and ambulatory care cost (24.4%). Pulmonary arterial hypertension (PAH) and gastrointestinal (GIT) involvement were the major determinants of healthcare cost (OR 2.3 and 1.8, *P* = .01 for hospitalizations; OR 2.8 and 2.0, *P* = .01 for ambulatory care; OR 7.8 and 1.6, *P* < .001 and *P* = .03 for medication cost, respectively). SSc is associated with substantial healthcare utilization and direct economic burden. The most costly aspects of SSc are PAH and GIT involvement.

## Abstract 2

Supplemental Digital Content is available in the text

## 1 Introduction

Systemic sclerosis (SSc) is a chronic multisystem autoimmune disease characterized by skin and internal organ fibrosis.^[1]^ It is classified into limited (lcSSc) and diffuse cutaneous (dcSSc) disease subtypes based on the extent of skin involvement. Worldwide prevalence of SSc varies from 7/million to 489/million,^[1]^ with the reported prevalence in Australia being one of the highest.^[2]^

Due to the progressive multiorgan nature of SSc long-term follow-up and a multidisciplinary approach to patient care are required. Therefore, it is not surprizing that SSc is one of the most costly rheumatic diseases, with SSc patients utilizing more healthcare dollars per annum than age and sex-matched counterparts with rheumatoid arthritis (RA), psoriatic arthritis, and/or inflammatory myopathies.^[3–5]^

Studies of healthcare utilization and economic burden specific to SSc are scarce and those available are cross-sectional or retrospective in nature, with small patient cohorts.^[6–8]^ Economic and health-related quality of life studies are essential for clinicians and health policy makers in order to quantify the burden of disease and to assess the outcomes of health policies and interventions.

All Australian citizens and permanent residents have access to public healthcare provided by a universal public health insurance scheme named Medicare Australia, established in 1984 by the Australian government. Medicare contributes to the costs of inpatient treatment in public hospitals, the payment of benefits for professional health services listed on the Medicare Benefits Schedule (MBS) including the cost of ambulatory care services, and subsidization of prescription medication cost under the Pharmaceutical Benefits Scheme (PBS).^[9]^ Under Medicare, the only out-of-pocket costs for patients are pharmacy dispensing fees for medications and gap fees for ambulatory care in a private setting where the amount charged by the healthcare provider exceeds the MBS rebate.

The Australian hospital system includes public hospitals, which are funded by the Australian government, and private hospitals, which are funded by private health insurance and compensation schemes. Public hospitals provide most of the emergency department (ED) services (94%), outpatient services (97%), and hospital admissions (60%). Although private hospitals provide predominantly same day admissions (69%) and elective surgery (66%), the complexity of hospital care provided to admitted patients is similar in both public and private hospitals.^[9]^ Patterns of healthcare utilization are similar across all Australian states.^[9]^

In order to determine the direct economic burden of SSc, we sought to quantify total healthcare utilization and associated cost in Australian SSc patients from the state of Victoria which accounts for approximately 25% of the Australian population.^[10]^

## 2 Method

Consecutive patients in Victoria prospectively enrolled in the Australian Scleroderma Cohort Study (ASCS), a multicenter study of risk and prognostic factors for cardiopulmonary and other clinically important outcomes in SSc, were included. The ASCS contains comprehensive demographic, disease-related and medication use data that are collected annually, and entered into a custom-made database. Written consent was obtained from all patients at recruitment and ethical approval was obtained from the participating hospitals (St Vincent's Hospital Melbourne and Monash Health Melbourne).

### 2.1 Inclusion and exclusion criteria

We included all adult (>18 years) SSc patients recruited between January 2011 and December 2015. Regardless of enrolment date during this time, all healthcare costs between 2011 and 2015 for each patient were included in the data linkage described below. Although the ASCS is a nationwide study, Victoria was chosen as it is Australia's most populous state, comprises the majority of patients enrolled in ASCS, and has the most complete clinical data entered in the database. Prior to 2013, all patients fulfilled either the American College of Rheumatology (ACR) or Leroy and Medsger criteria for SSc,^[11,12]^ while post 2013 all patients were reclassified according to the 2013 ACR/EULAR classification criteria.^[13]^

### 2.2 Healthcare utilization

Healthcare utilization in Victoria was captured by means of data linkage. Through the Australian Institute of Health and Welfare, the ASCS database of deidentified SSc patients’ demographic information, disease-related data, medication, and patient reported outcome measure data were merged with the Victorian Admitted Episodes Dataset, the Victorian Emergency Minimum Dataset, and the MBS, thereby capturing all hospital admissions, ED presentations, and ambulatory care use. All merged data were deidentified prior to being stored and analyzed within the Secure Unified Research Environment, which is a remote-access secure computing environment that allows researchers to analyze linked data. As the Australian administrative databases are not linked across the country, completing a nationwide data linkage approach across states other than Victoria was not feasible.

### 2.3 Hospitalization and ED presentation data

The Victorian Admitted Episodes Dataset contains information on the primary diagnosis and/or procedure for patients admitted to hospital, their length of stay (LOS), hours in the intensive care unit and/or coronary care unit, and the diagnosis related group (DRG) for each admission. The Victorian Emergency Minimum Dataset contains information on the primary diagnosis and/or procedure for ED presentation, the triage category, and departure destination.

We divided our admitted and ED presentation patients into groups: low and high number of admissions; ED presentations; and LOS, based on the median number of admissions, ED presentations, and LOS, respectively, for the whole group. We then compared demographics and disease-related data in the low versus high groups.

### 2.4 Ambulatory care data

The MBS lists the professional services for which a government-funded payment can be claimed^[14]^ and includes GP visits, physician and surgical visits, and allied health visits, together with pathology and imaging, performed within Australia. Every MBS-listed service is assigned a “schedule fee,” an amount the government considers appropriate for the service and a “benefit payable fee,” that is approximately 75% to 85% of the schedule fee which the government contributes. Some practitioners’ fees are above the benefit payable fee meaning that patients pay an “out of pocket fee” that is not redeemable from the government. This out of pocket fee was not calculated in this paper. Furthermore, any services not covered by the MBS that patients utilize and self-fund, such as osteopathy, were not captured.

The MBS does not cover public hospital outpatient clinics. To avoid underestimating ambulatory care utilization, we also estimated the outpatient clinic cost of SSc patients enrolled in ASCS at St Vincent's Hospital Melbourne by quantifying use and associated costs in collaboration with the decision support unit at the hospital. At Monash Health, all rheumatology outpatient visits are “privately” billed, and hence use and associated cost are captured in the MBS database.

### 2.5 Medication data

Medication utilization and duration of use was determined from the ASCS database, wherein detailed medication use data are recorded at each visit for all patients enrolled. Although the Australian government PBS database records medications dispensed to individuals covered by Medicare, only medications with costs above a threshold of Australian Dollar (AUD)$38.80 are recorded in this administrative database. Furthermore, ethical approval is not granted by the data custodians for linkage with both the PBS and MBS databases due to concerns regarding potential patient reidentification with merging of multiple datasets.

### 2.6 Costing methodology

#### 2.6.1 Hospital cost

In the Australian public hospital system, every admission is allocated a DRG, based on the International Classification of Diseases (ICD) and a weighted inlier equivalent separation (WIES) based on the primary reason for admission, DRG, complications, LOS, and individual patient factors. The WIES value incorporates the cost of investigations and medications administered for that condition in a hospital setting. Cost was calculated based on financial year of admission and the corresponding WIES for that financial year. A sensitivity analysis of hospitalization related cost was performed based on estimated private hospitalizations in the same period (40%) on the premise that the patient complexity was the same between public and private admissions.^[9]^

Victorian ED presentations are “block funded,” meaning that each hospital receives an annual amount of money that is calculated based on anticipated ED presentations and cost of staff required to maintain ED cubicles and patient care. For the purpose of this paper, the average cost of an ED presentation during this time period (2011–2015) was calculated for each of the 2 participating Victorian hospitals (AUD$689.57 for St Vincent's Hospital and AUD$590.00 for Monash Health).

#### 2.6.2 Ambulatory care cost

Ambulatory care cost was calculated using the total MBS “benefit payable fee” as this is what the government contributed toward each service, making it an accurate reflection of the direct cost and hence economic burden to the Australian government.

#### 2.6.3 Medication cost

Medication cost was determined from the PBS dispensed price for maximum quantity (DPMQ) paid for the standard dose of each medication. The DPMQ is the cost the government contributes toward each medication dispensed, thus making the medical cost an accurate reflection of the direct cost to the Australian government. Average duration on each medication was calculated and the cost of a standard dose of each medication to the government was estimated. All medications with a PBS listing were included in the calculation of total medication cost, with only complementary and over the counter medications excluded.

### 2.7 Extrapolation of cost to all Australian SSc patients

The proportion of SSc patients recruited in the ASCS was estimated relative to the prevalence of SSc in Australia (21.1 per 100,000)^[15,16]^ and population data from the Australian Bureau of Statistics (Victorian population of 5,996,400, Australian population of 24,304,682 in 2015).^[10]^ Extrapolation of total cost to the Australian government was based on the estimated number of SSc patients in Victoria (n = 1266) and in Australia (n = 5129).^[15,16]^ Costs are also presented in US dollars (USD) based on currency conversion performed on 10th May 2017.

### 2.8 Clinical data

Patient demographics and clinical variables were obtained from the ASCS database. Prior to the ASCS clinical patient data linkage, any missing patient data including disease manifestations, medication utilization, and patient status were retrieved from the treating clinician and recorded before the linkage occurred. As patient data are collected in the ASCS on an annual basis, patients were classified as lost-to-follow-up if they had been uncontactable for a period of 18 months. The period of 18 months was selected to allow for delays in data entry. There were 2 patients out of 531 Victorian SSc patients lost-to-follow-up included in this data linkage study. Clinical manifestations and autoantibody status were defined as present if present ever from the time of diagnosis of SSc. Extent of skin involvement was determined using the modified Rodnan skin scores (mRSS). Physician classification of patients into lcSSc and dcSSc was confirmed by reviewing recorded mRSS scores. lcSSc was defined as skin involvement distal to the elbows and knees with or without facial involvement; dcSSc was defined as skin involvement proximal to the elbows and knees, with or without truncal involvement. Pulmonary arterial hypertension (PAH) was diagnosed on right heart catheterization (RHC) according to international criteria.^[17]^ Interstitial lung disease was defined based on characteristic changes on high-resolution computer tomography lung. Renal crisis was defined as a combination of any 2 of the following criteria: new onset severe hypertension without an alternate etiology; microangiopathic hemolytic anemia; and rising creatinine. Upper gastrointestinal (GIT) involvement included the presence of reflux esophagitis and/or esophageal stricture on endoscopy. Lower GIT involvement included the presence of bowel dysmotility defined on barium and nuclear medicine studies, antibiotic response and/or characteristic symptoms, and fecal incontinence. GIT involvement included the presence of both upper and lower GIT involvement. Hand dysfunction was defined based on the presence of joint contractures and/or deformities.

Global disease activity and severity were measured using physician-rated scales ranging from 0 to 10, with 0 being no activity or severity and 10 being very severe activity or severity.^[18]^

### 2.9 Statistical analysis

Data are presented as mean ± standard deviation for normally distributed and median (interquartile range [IQR]) for nonnormally distributed continuous variables, and as number (percentage) for categorical variables. Differences in frequency were tested using chi-square and Fisher exact tests. Univariable and multivariable logistic regression were used to determine the associations of different factors with healthcare utilization and cost. A 2-tailed *P* value of.05 or less was considered statistically significant. All statistical analyses were performed using STATA 14.0 (StataCorp LP, College Station, TX).

## 3 Results

### 3.1 Hospitalizations

Of the 531 Victorian SSc patients enrolled in ASCS between January 2011 and December 2015, 432 patients (81.4%) were admitted to hospital at least once. The total number of hospital admissions during this period was 3948, equating to an average of 789.9 admissions annually. The median (IQR) number of hospitalizations per patient per year was 5 (2–11) with a median LOS of 1 day (1–2.5). There was no difference in admission frequency among the seasons or months of the year. Of the admitted patients, 70 (16.2%) patients spent time in the intensive care unit (median hours 47 [25–124]) and 42 (9.7%) spent time in the coronary care unit (median hours 31 [6–59.5]).

The majority of admissions were acute care (96.9%), followed by rehabilitation (0.9%), palliative care (0.7%), geriatric (0.7%), and acute mental health service (0.7%).

Patient characteristics by admission status are summarized in Table 1. Factors associated with hospital admission in univariable and multivariable logistic regression analysis are summarized in Supplementary Table 1, while factors associated with above average number of hospital admissions and LOS, determined using univariable and multivariable logistic regression analysis, are shown in Supplementary Tables 2 and 3.

**Table 1:**
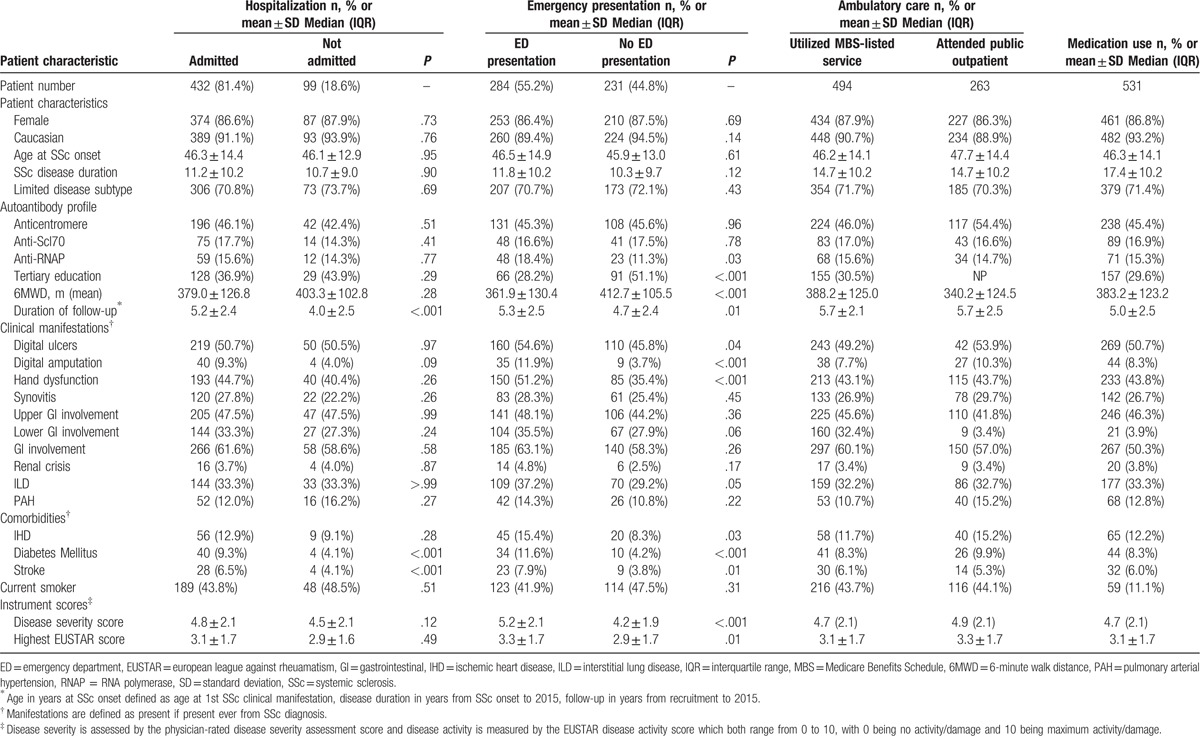
Patient characteristic by health care utilization.

The most common principal diagnoses for hospital admissions in SSc patients hospitalized between 2011 and 2015 are summarized in Supplementary Table 4. Disorders of the digestive system accounted for the majority of major admission categories (21.8%), followed by disorders of the musculoskeletal system (MSK) and connective tissue accounting for 21.3% of admissions (Supplementary Table 5).

### 3.2 Hospital admission cost

The cost associated with inpatient hospitalization during 2011 to 2015 for Victorian SSc patients within ASCS amounted to AUD$12,734,394.95 (USD$9,372,514.68) (annual cost of AUD$2,546,878.99 [USD$ 1,874,502.94]). The median hospitalization cost per patient over the 5-year period was AUD$16,144.5 (IQR 5,963.16–43,754.14 [USD$11,882.35, IQR 4388.89–32,203.05]), amounting to an annual per patient median cost of AUD$3228.9 (USD$2376.47).

Factors associated with above median hospitalization costs, determined using univariable logistic regression analysis, are summarized in Supplementary Table 6. Factors associated with above median hospitalization costs, determined using multivariable logistic regression analysis, included PAH (OR 2.3, *P* = .01), digital amputation (OR 4.1, *P* = .01), longer SSc disease duration (OR 1.0, *P* = .02), renal crisis (OR 3.2, *P* = .04), upper GIT involvement (OR 1.8, *P* = .01), and coexistent diabetes mellitus (OR 3.6 *P* < .001) (Table 2).

**Table 2:**
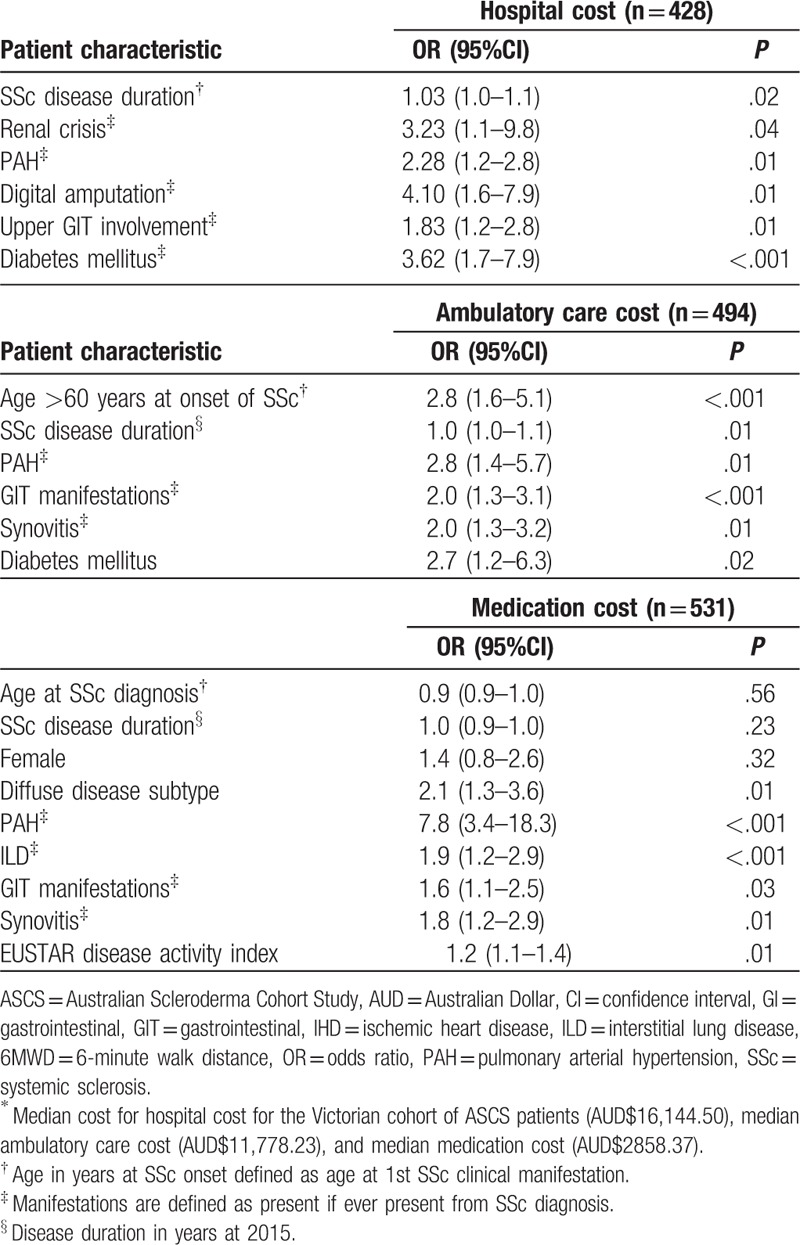
Independent determinants of above median health care cost^∗^ by multivariable logistic regression analysis.

### 3.3 Emergency department presentations

Of the 531 Victorian SSc patients enrolled in ASCS between January 2011 and December 2015, 293 patients (55.2%) presented to the ED at least once. The total number of ED presentations during this period was 1455 (1090 to St Vincent's Hospital and 365 to Monash Health, Melbourne), equating to an average of 291 presentations per year. The median number of ED presentations per patient per year was 3 (IQR 1–5) with no difference among the seasons or months of the year.

Of those patients who presented to the ED, all presentations were emergency presentations (99.7%) except for one patient who had a planned return to the ED. Patient characteristics by ED presentation are summarized in Table 1. Factors associated with ED presentation, determined using univariable, and multivariable logistic regression analysis, are shown in Supplementary Table 1. Factors associated with above median annual number of ED presentations (n = 3) in SSc, determined using univariable logistic regression analysis are shown in Supplementary Table 2. Factors associated with above median annual number of ED presentations in SSc, determined using multivariable logistic regression analysis, included the presence of digital ulcers (OR 1.7, *P* = .01), lower 6-minute walk distance (OR 0.9, *P* = .01), and coexistent diabetes mellitus (OR 2.9, *P* = .02) (Table 2).

The most common principal diagnoses for ED presentations in SSc patients are summarized in Supplementary Table 4. Chest pain (5.9%) was the most common principal diagnosis for ED presentation followed by abdominal pain (3.5%).

### 3.4 ED presentation cost

The cost associated with ED presentations during 2011 to 2015 for SSc patients within ASCS amounted to AUD$961,671.30 (USD$707,789.87) (average annual cost AUD$192,334.26 [USD$141,557.97]). No sensitivity analysis was performed to account for ED presentations to private hospitals, as over 94% of ED presentations in Australia occur in public hospitals.

### 3.5 Total hospital costs

Total hospital cost, including hospitalization and ED costs, for all Victorian SSc patients between 2011 and 2015 amounted to AUD$32,653,898.06 (USD$ 24,033,268.47) (average annual cost of AUD$6,530,779.61, USD$4,806,653.69). For all Australian SSc patients, this would amount to AUD$132,292,135.17 (USD$97,376,009.45) (average annual cost of AUD$26,458,427.04, USD$19,473,401.90), with an annual cost per patient of AUD$5158.59 (USD$3796.72).

Assuming that public hospital use accounted for only 60% of all hospital use, total hospital costs extrapolated to all Australian SSc patients over the 5 year period would be AUD$220,486,891.80 (USD$162,278,303.97), with an average annual cost of AUD$44,097,378.39 (USD$32,455,660.79) and an annual cost per patient of AUD$8597.66 (USD$6327.87).

### 3.6 Ambulatory care utilization

Of the 531 Victorian SSc patients enrolled in the ASCS between 2011 and 2015, 494 (93.0%) utilized an ambulatory care service reimbursed through the MBS. Patient characteristics are summarized in Table 1.

The majority of ambulatory care cost for ASCS SSc patients was for professional attendances (MBS category 1 services) totaling AUD$2,649,682.35 between 2011 and 2015. Diagnostic procedures (MBS category 5), encompassing the use of ultrasound, echocardiography, computer tomography, magnetic resonance imaging, and nuclear medicine, comprised the 2nd most costly service category, totaling AUD$1,386,737.90, followed closely by pathology services (MBS category 6) totaling AUD$1,070,187.00 and therapeutic procedures (MBS category 3) totaling AUD$926,009.25. Table 3  summarizes all MBS services provided, their associated frequency of use and costs.

**Table 3:**
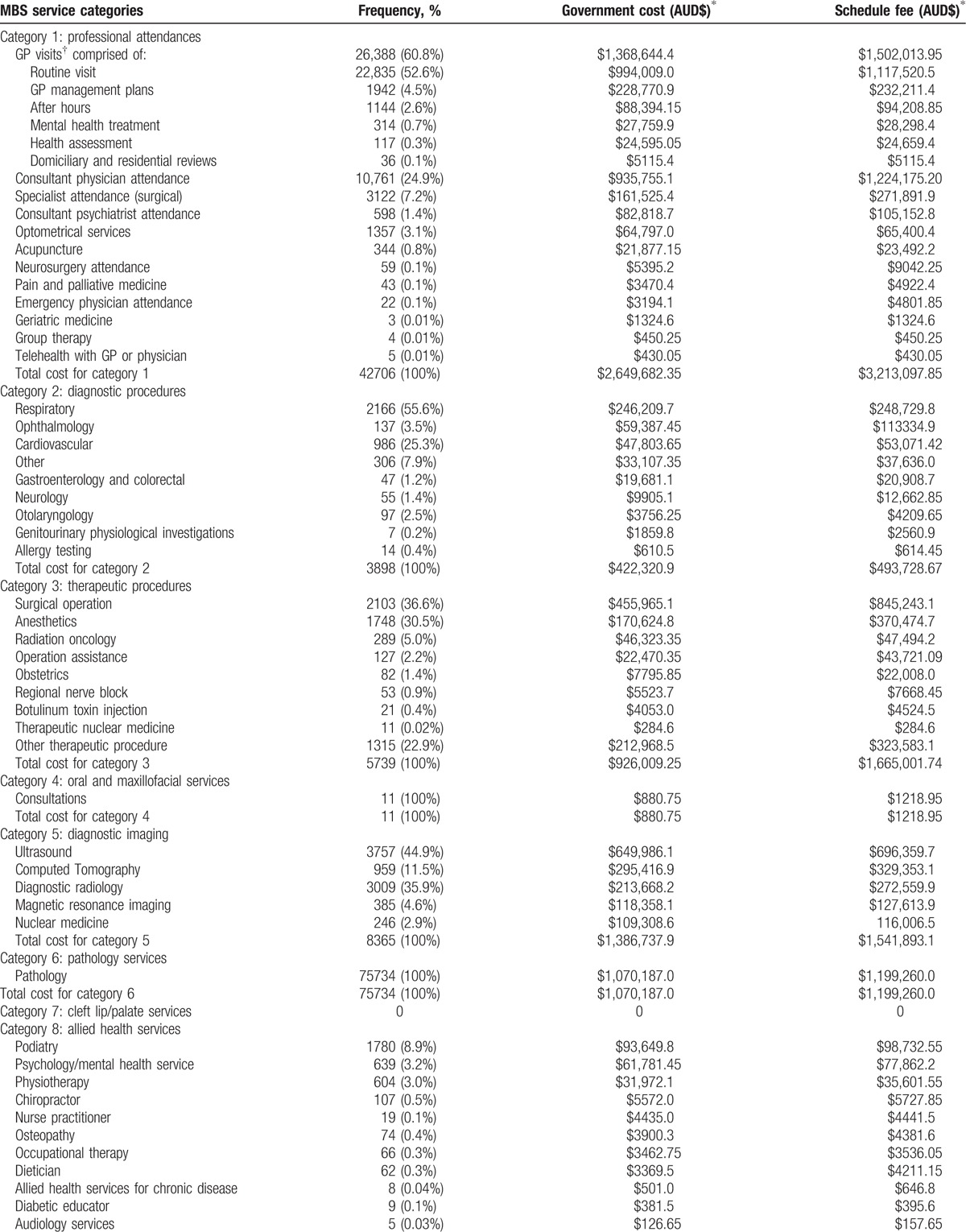
Frequency and cost of MBS services provided between 2011 and 2015 (“benefits paid”).

**Table 3 (Continued):**
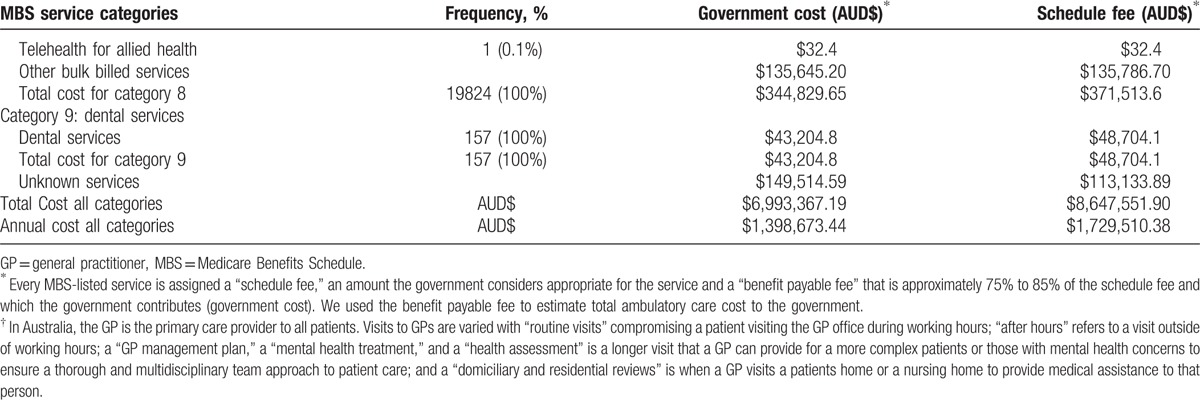
Frequency and cost of MBS services provided between 2011 and 2015 (“benefits paid”).

Between 2011 and 2015, allied health services (MBS category 8) accounted for the 6th most expensive MBS service for ASCS SSc patients at AUD$344,829.65. Podiatry was the most frequently used and costly service accounting for 8.9% of all allied health services and AUD$93,649.80, followed by psychological therapy (3.2%, AUD$61,781.45).

### 3.7 MBS-related ambulatory cost

The total MBS cost of ambulatory care for Victorian ASCS SSc patients over this 5-year period was AUD$6,993,367.19 (USD$5,147,118.25) (average annual cost of AUD$1,398,673.44 (USD$1,029,423.65), annual per patient cost of AUD$2831.32 [USD$2083.85]).

### 3.8 Determinants of ambulatory care cost

Patient characteristics based on the median total ambulatory care cost (AUD$11,778.23) are shown in Supplementary Table 7. Patient factors associated with above median ambulatory care costs, determined using univariable logistic regression analysis, are presented in Supplementary Table 6. Factors associated with above the median ambulatory care costs, determined using multivariable logistic regression analysis, included age over 60 years at SSc onset (OR 2.8, *P* =  < .001), longer SSc disease duration (OR 1.0, *P* = .01), PAH (OR 2.8, *P* = .01), GIT involvement (OR 2.0, *P* < .001), synovitis (OR 2.0, *P* = .01), and coexistent diabetes mellitus (OR 2.7 *P* = .02) (Table 2).

### 3.1 Public hospital outpatient clinic cost for SSc patients

Over the same 5-year period as described above, 263 Victorian SSc patients enrolled in the ASCS attended the outpatient clinic at St Vincent's Hospital, a large tertiary public hospital in Melbourne, Victoria. Patient characteristics were similar to the MBS merged dataset and are described in Table 1. Outpatient clinic attendance and associated costs are outlined in Table 4.

**Table 4:**
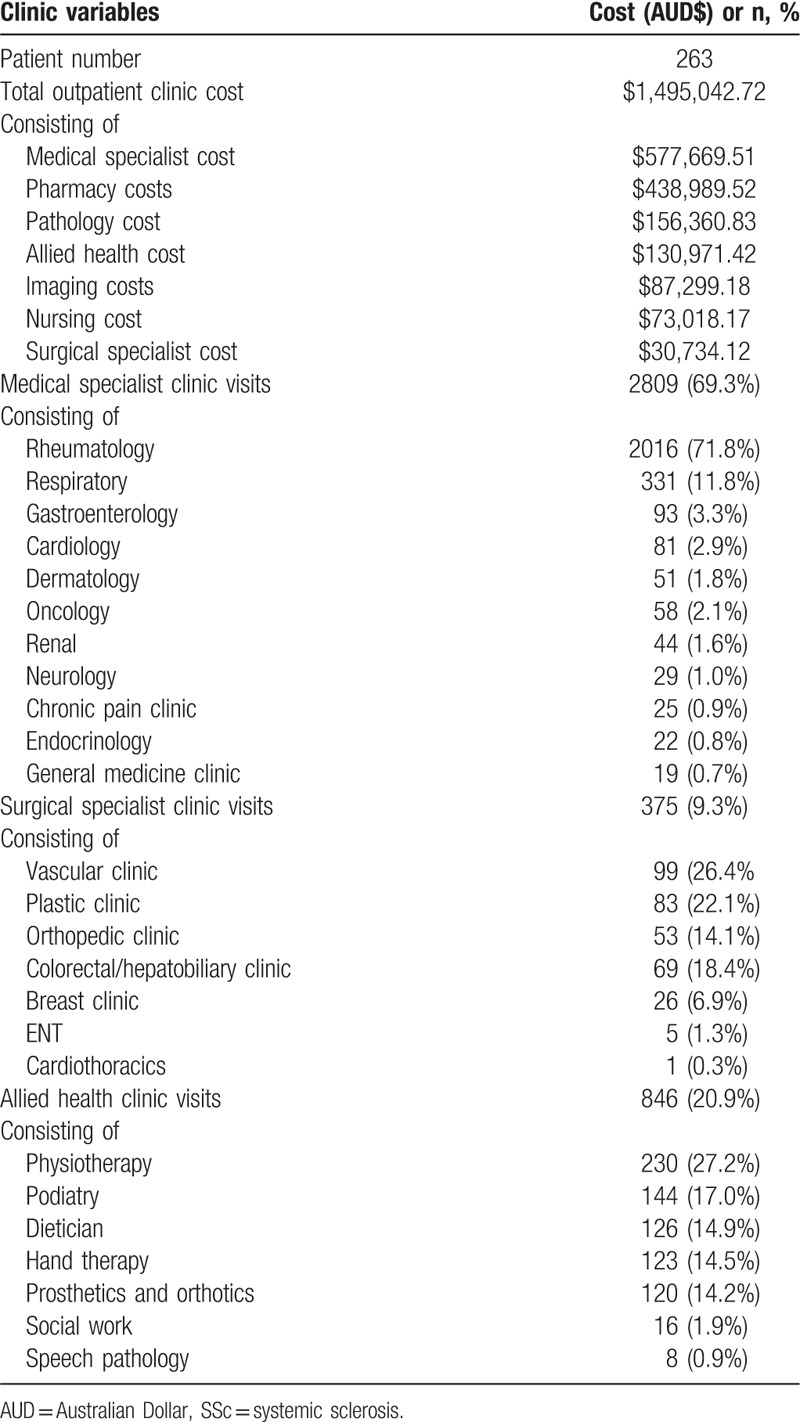
Public tertiary hospital outpatient clinic costs associated with SSc patients.

The total cost for St Vincent's hospital outpatient clinic over this 5-year period was AUD$1,495,042.72 (USD$1,100,351.44) (annual cost of AUD$299,008.54 (USD$220,070.29), annual per patient cost of AUD$1136.92 [USD$836.77]). This cost encompassed medical, surgical, and allied health costs in addition to pathology and imaging costs. As mentioned above, rheumatology outpatient clinics at Monash Health are privatized and their cost is captured in the MBS cost. Therefore, outpatient clinic cost has not been extrapolated to all Victorian or Australian SSc patients due to the potential for over-estimation.

### 3.2 Medication utilization

Medications were categorized by their clinical indication and SSc disease manifestations as shown in Table 5. From 2011 to 2015, the total medication cost in Victorian SSc patients was AUD$9,603,557.77 (USD$7,068,218.52) with an annual cost per patient of AUD$3617.16 (USD$2662.23).

**Table 5:**
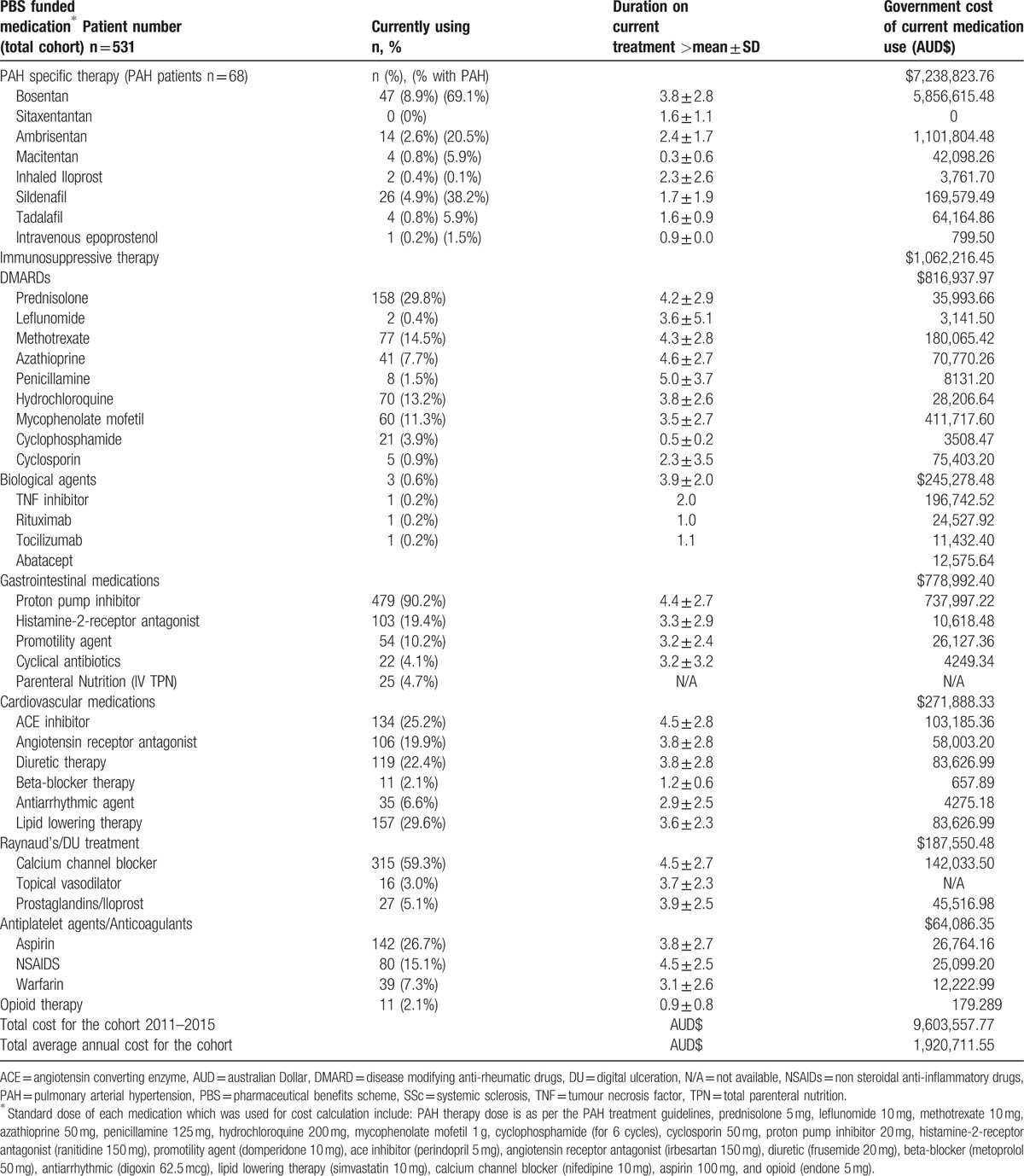
Frequency and cost of PBS medications used by SSc patients between 2011 and 2015.

PAH specific therapies accounted for the highest medication-related cost (AUD$7, 238,823.76, USD$5,327,774.29), despite only 10.7% of patients in the cohort having PAH. The annual medication cost per PAH patient was AUD$21,290.66 (USD$15,669.93). Immunosuppressive therapies were the 2nd most costly medication class accounting for a cost of AUD$1,062,216.45 (USD$781,791.31) between 2011 and 2015 (average annual cost of AUD$212,443.29, USD$156,358.26).

### 3.3 Determinants of medication cost

Patient characteristics by median medication cost for the cohort (AUD$2858.369) are shown in Supplementary Table 7. Factors associated with above median medication costs in univariable logistic regression analysis are presented in Supplementary Table 6. Factors associated with above median medication cost, determined using multivariable logistic regression analysis, included dcSSc (OR 2.1, *P* = .01), PAH (OR 7.8, *P* < .001), interstitial lung disease (OR 1.9, *P* < .001), GIT manifestations (OR 1.6, *P* = .03), synovitis (OR 1.8, *P* = .01), and higher SSc disease activity score (OR 1.2, *P* = .01) (Table 2).

### 3.4 Total health care cost to the Australian government

Extrapolating total health care cost from 2011 to 2015 to all Australian SSc patients would equal AUD$297,663,404.77 (USD$219,080,200.42) (annual cost AUD$59,532,680.95, USD$43,816,040.08), with an annual cost per patient of AUD$11,607.07 (USD$8542.80). Hospital costs accounted for the majority of these costs (44.4% of total annual cost, AUD$26,458,427.04, USD$19,473,401.90), followed by medication cost (31.2%, AUD$18,552,413.60, USD$13,654,576.41), and ambulatory care cost (24.4%, AUD$14,521,840.30 (USD$10,688,074.46) (Table 6).

**Table 6:**
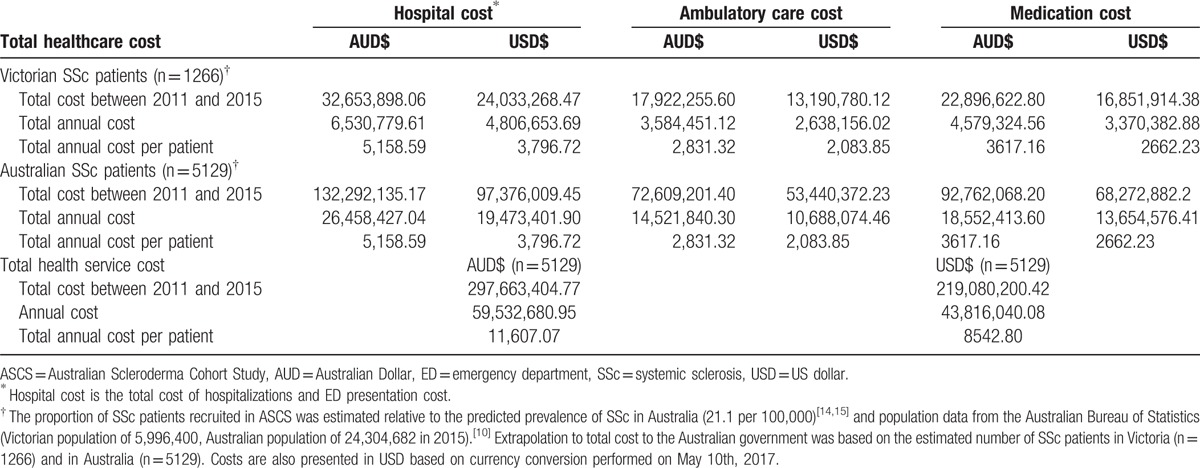
Total healthcare cost in SSc.

## 4 Discussion

Our study demonstrates that despite SSc being a low prevalence condition, it is associated with substantial healthcare utilization and cost. In our Victorian SSc cohort, over 80% (81.4%) were admitted to hospital at least once between 2011 and 2015, totaling 3948 hospital admissions with an overall cost of AUD$12,734,394.95 (USD$9,372,514.68); over half (55.2%) presented to the ED on at least 1 occasion, totaling 1455 presentations with an associated cost of AUD$192,334.26 (USD$141,557.97) and 93.0% utilized an ambulatory care service reimbursed through the MBS AUD$17,922,255.60 (USD$13,190,780.12). Using SSc prevalence data, the total health care expenditure extrapolated to all Australian SSc patients during this time period was AUD$297,663,404.77.15 (USD$219,080,200.42) (annual cost AUD$59,532,680.95, USD$43,816,040.08) with an annual cost per patient of AUD$11,607.07 (USD$8542.80).

MSK conditions are increasingly recognized as a significant contributor to the global burden of disease (GBD) constituting the 2nd most common cause of disability worldwide.^[19]^ In Australia, arthritis and MSK conditions (including osteoarthritis, RA, back problems, and “other” MSK conditions such as SSc) constitute the 4th highest healthcare expenditure in 2008 to 2009 consuming 8.7% of the AUD$65,129 million healthcare expenditure allocated to disease groups.^[20]^ The financial healthcare burden including hospital admitted patient services, out-of-hospital medical services, and prescription medications associated with MSK conditions in Australia was AUD$1637 million for osteoarthritis, AUD$1177 million for back problems, AUD$44 million for RA, and AUD$44 million for osteoporosis.^[20]^ There have been no previous studies assessing the cost of SSc in Australia, but based on our results, the annual cost of SSc (AUD$59 million) is more than some other MSK disorders with higher prevalence, for example, osteoporosis.^[20]^

SSc-specific healthcare-related costs vary geographically and depending on what is included in the cost estimation (hospitalization, ambulatory care, and/or medication cost). Within Australia, for example, access to certain emerging treatment opportunities for the treatment of SSc, such as autologous stem cell transplantation in SSc,^[21,22]^ is not equitable between all Australian States being currently only available at 1 tertiary institution in New South Wales (NSW). Consequently, the costs of SSc in Victoria may vary from that in NSW. Our estimated direct cost per SSc patient per annum is AUD$11,607.07 (USD$8542.80), which is remarkably similar to estimate worldwide. For example, the annual direct per patient cost of SSc in Canada is estimated to be CAD$5038,^[5]^ in the US to range between USD$8441 and $17,365,^[23,24]^ in Spain to be €8235,^[7]^ in France to be €8452,^[6]^ in Hungry to be €4232,^[3]^ and in Europe to range between €1413 and 17,300.^[8]^ In our study, hospital related cost accounted for the majority of the total direct healthcare cost (44.4%), followed by medication cost (31.2%) and ambulatory care cost (21.1%). Our healthcare costs in SSc are consistent with other countries such as Canada where hospital cost accounted for 34.9% while medication cost accounted for 31.2% of direct cost in SSc patients,^[5]^ and in America were ambulatory care accounted for 19% of the total healthcare cost incurred by SSc patients.^[25]^

Utilization of ambulatory care, particularly allied health services, among Australian SSc patients was very common in our study and is mirrored in other countries such as the Netherlands where 61% of the SSc cohort had utilized allied health services within the last year most commonly physiotherapy, and averaged 7.5 visits annually to an allied health provider.^[26,27]^ These results reinforce the importance of a multidisciplinary team approach to SSc management and highlight the need for consistent government funding to ensure these services remain accessible to all SSc patients regardless of their socioeconomic background.

SSc-PAH contributes to significant morbidity, mortality, and poor health-related quality of life compared with SSc patients without PAH.^[28–30]^ Our study shows that that the presence of SSc-PAH is associated with a higher frequency of inpatient hospitalization, longer LOS, and higher hospital costs, in addition to a higher frequency of ambulatory service utilization and associated cost. Furthermore, despite only 10.7% of patients having SSc-PAH in our study, PAH specific therapy accounted for the majority (75.4%) of the total medication-related cost. This highlights the significant and concerning burden associated with SSc-PAH impacting not only patients but also the wider society through healthcare utilization and associated cost. Further research in this area is required to enable effective intervention to modify this burden.

Diabetes mellitus (DM) affects over 1 million Australians and is associated with a significant direct healthcare cost,^[31]^ which is why we think that the coexistence of DM was an independent determinant of healthcare costs in SSc patients.

We expect our findings to be generalizable to other developed countries with a similar healthcare system. Our results highlight that “other” MSK conditions such as SSc are not only an important contributor to the burden of disease but are also a central component of health expenditure in many high-income and middle-income countries.^[20,32]^

Strengths of our study include the large, well-defined multicenter cohort of patients with confirmed SSc, and detailed patient-related data recorded systematically using well-validated tools and methods. Furthermore, our healthcare cost is a true reflection of the government cost derived from the MBS benefit paid and PBS DPMQ price. This study was performed to quantify the direct healthcare costs of Australian SSc patients. We did not evaluate the economic implications of treating certain disease manifestations such as PAH and/or GIT manifestations. We do note the importance of such economic studies and recommend this as an area of further research. Limitations include the possibility of underestimating the true cost associated with hospitalizations due to geographic variation across Australian States, which may impact on treatment opportunities (such as ASCT). We were also unable to determine if there was a relationship between the geographic region and healthcare utilization due to ethical restrictions with potentially reidentifying variables such as patients’ and/or hospitals’ geographic postcode. Additionally, hospitalizations in the private sector which were not included, although we attempted to estimate the additional private sector hospitalization costs in our sensitivity analyses. Furthermore, we did not include the cost of public outpatient clinics, any privately funded allied health service not covered by the MBS, or medications or treatment, including parenteral nutrition not subsidized by the PBS (Table 5).

## 5 Conclusion

This data linkage study provides the first comprehensive estimate of healthcare use and its costs in SSc patients in Australia, and is the largest study of its kind to date. We believe our findings are applicable to other developed countries with a similar standard of healthcare. SSc is associated with a substantial direct cost burden. Economic studies such as ours will allow the development of tailored policies to improve patient care and associated costs, and reduce the long-term impact of this devastating disease.

## Acknowledgments

The authors thank all the investigators of the Australian Scleroderma Interest Group (ASIG): C Hill, Adelaide, South Australia; S Lester, Adelaide, South Australia; P Nash, Sunshine Coast, Queensland; G Ngian, Melbourne Victoria; M Nikpour, Melbourne, Victoria; S Proudman, Adelaide, South Australia; M Rischmueller, Adelaide, South Australia; J Roddy, Perth, Western Australia; J Sahhar, Melbourne, Victoria; W Stevens, Melbourne, Victoria; G Strickland, Geelong, Victoria; V Thakkar, Liverpool, New South Wales; J Walker, Adelaide, South Australia; and J Zochling, Hobart, Tasmania.

## Supplementary Material

Supplemental Digital Content
