# Supplementary material for: Quantifying the direct public health care cost of systemic sclerosis: A comprehensive data linkage study
Source: Medicine (Baltimore). 2017 Dec 1;96(48):e8503. doi: 10.1097/MD.0000000000008503 (PMC5728733; doi:10.1097/MD.0000000000008503)
Supplement: Supplemental Digital Content [file medi-96-e8503-s001.docx]

Supplementary Table 1 Determinants of hospital admission and ED presentation in univariable and multivariable logistic regression analysis

|  | **Determinants of hospital admission** | | **Determinants of ED presentation** | |
| --- | --- | --- | --- | --- |
| **Characteristic** | **OR (95%CI)** | **p-value** | **OR (95%CI)** | **p-value** |
| **Univariable analysis** |  |  |  |  |
| Patient characteristic  Female  Age at onset of SSc^#^  Tertiary education  Diffuse disease  6MWD, m | 0.89 (0.5-1.7)  1.00 (0.9-1.0)  0.75 (0.4-1.3)  1.14 (0.7-1.9)  0.9 (0.9-1.0) | .73  .92  .29  .61  .17 | 1.00 (0.9-1.0)  0.9 (0.5-1.5)  0.37 (0.3-0.6)  1.05 (0.7-1.5)  0.99 (0.9-0.9) | .47  .69  < .001  .81  < .001 |
| Clinical Manifestations*  Digital Ulcers  Digital amputation  Hand dysfunction  Lower GI involvement  GI involvement  Renal Crisis  ILD  PAH | 1.01 (0.7-1.6)  2.42 (0.9-6.9)  1.19 (0.8-1.9)  1.49 (1.0-2.2)  1.46 (1.0-2.2)  0.9 (0.3-2.8)  1.00 (0.6-1.6)  0.71 (0.4-1.3) | .97  .09  .44  .04  .04  .88  < .99  .27 | 1.42 (1.0-2.0)  3.48 (1.6-7.4)  1.91(1.4-2.7)  1.42 (0.9-2.1)  1.22 (0.9-1.7)  1.96 (0.7-5.2)  1.44 (0.9-2.1)  1.38 (0.8-2.3) | .04  < .001  < .001  .06  .26  .17  .05  .22 |
| Co-morbidities*  IHD  Diabetes Mellitus  Stroke  Current smoker | 1.49 (0.7-3.2)  2.21 (0.8-6.4)  1.47 (0.5-4.3)  0.83 (0.5-1.3) | .29  .14  .48  .39 | 2.01 (1.2-3.5)  2.91 (1.4-6.0)  2.09 (0.9-4.6)  0.79 (0.6-1.2) | .01  .01  .06  .18 |
| **Multivariable analysis** | **OR (95%CI)** | **p-value** | **OR (95%CI)** | **p-value** |
| Tertiary education  Digital amputation*  ILD*  Diabetes Mellitus*  Lower GIT involvement* | 0.9 (0.5-1.5)  2.3 (0.8-6.5)  0.9 (0.6-1.6)  1.9 (0.7-5.7)  1.4 (0.9-2.3) | .18  .34  .12  .25  .37 | 0.37 (0.2-0.6)  3.69 (1.5-9.5)  1.56 (1.0-2.4)  3.14 (1.3-7.5)  1.49 (0.9-2.4 | < .001  .01  .04  .01  .05 |

Abbreviations: gastrointestinal (GI), pulmonary arterial hypertension (PAH), interstitial lung disease (ILD), six minute walk distance (6MWD), ischemic heart disease (IHD)

* clinical manifestations are defined as present if ever present from SSc diagnosis

# age in years at SSc onset defined as age at first SSc clinical manifestation

Supplementary Table 2 Determinants of above median number of hospital admissions^, length of stay^ and ED presentations^ by univariable logistic regression analysis

| **Determinants of above average:** | **number of hospital admissions**  **(n=432)** | | **hospital length of stay**  **(n=432)** | | **number of ED presentations**  **(n=293)** | |
| --- | --- | --- | --- | --- | --- | --- |
| Variable | OR (95%CI) | p-value | OR (95%CI) | p-value | OR (95%CI) | p-value |
| Patient characteristic  Female  Age at onset of SSc^#^  Tertiary education  Diffuse disease subtype  SSc disease duration^#^  6MWD, m  Private health insurance | 1.15 (0.7-1.9)  1.01 (0.9-1.0)  0.72 (0.5-1.1)  1.16 (0.8-1.8)  1.01 (0.9-1.0)  0.9 (0.9-1.0)  0.8 (0.5-1.1) | .63  .06  .13  .48  .09  < .001  .17 | 0.82 (0.5-1.4)  1.01 (0.9-1.0)  0.58 (0.4-0.9)  1.04 (0.7-1.6)  1.0 (0.9-1.0)  0.99 (0.9-0.9)  0.58 (0.4-0.9) | .49  .02  .02  .85  .24  < .001  .01 | 1.04 (0.5-2.0)  1.01 (0.9-1.0)  0.57 (0.3-1.0)  1.12 (0.7-1.9)  1.01 (0.9-1.0)  1.01 (0.6-1.7)  NP | .91  .06  .06  .67  .17  .01  NP |
| Clinical manifestations*  Digital Ulcers  Digital amputation  Hand dysfunction  Upper GI involvement  Lower GI involvement  GI involvement  Renal Crisis  ILD  PAH | 1.72 (1.2-2.5)  2.95 (1.4-6.1)  2.17 (1.5-3.2)  1.59 (1.1-2.3)  1.49 (1.0-2.2)  1.49 (1.0-2.2)  1.73 (0.6-4.9)  1.32 (0.9-1.9)  2.31 (1.3-4.3) | .01  .01  < .001  .01  .04  .04  .29  .17  .01 | 1.69 (1.1-2.5)  3.39 (1.7-6.7)  1.24 (0.8-1.8)  1.13 (0.8-1.7)  0.89 (0.6-1.4)  0.82 (0.6-1.2)  1.09 (0.4-3.1)  1.08 (0.7-1.6)  2.82 (1.6–5.1) | .01  < .001  .29  .54  .62  .32  .88  .72  < .001 | 1.37 (0.9-2.2)  1.45 (0.7-2.9)  1.25 (0.8-1.9)  1.05 (0.7-1.7)  1.82 (1.1-2.9)  1.34 (0.8-2.2)  1.79 (0.6-5.3)  1.25 (0.8-2.0)  1.09 (0.6-2.1) | .18  .31  .35  .84  .01  .24  .29  .36  .79 |
| Co-morbidities*  IHD  Diabetes Mellitus  Stroke  Current smoker | 2.01 (1.1-3.6)  2.33 (1.2-4.7)  3.4 (1.4-8.1)  0.91 (0.6-1.3) | .02  .02  .01  .64 | 1.29 (0.7-2.3)  1.51 (0.8-2.9)  1.35 (0.6-2.9)  1.39 (0.9-2.1) | .89  .22  .45  .09 | 2.21 (1.2-4.2)  3.07 (1.4-6.6)  1.74 (0.7-4.1)  1.22 (08-1.9) | .01  .01  .21  .40 |

Abbreviations: gastrointestinal (GI), pulmonary arterial hypertension (PAH), interstitial lung disease (ILD), six minute walk distance (6MWD), ischemic heart disease (IHD), variable not provided in the dataset (NP)

^ average number of hospitalizations 5(2-11), average length of stay (LOS) in hospital 1(1-2.5), average number of ED presentations (n=3)

* manifestations are defined as present if ever present from SSc diagnosis

# age in years at SSc onset defined as age at first SSc clinical manifestation

Supplementary Table 3 Independent determinants of above median number of hospital admissions^, length of stay^ and ED presentations^ by multivariable logistic regression analysis

| **Patient characteristic** | **above average number of hospital admissions** | |
| --- | --- | --- |
|  | **OR (95%CI)** | **p-value** |
| Age at onset of SSc^#^  PAH*  Digital ulcers*  Upper GIT involvement*  Hand dysfunction*  Stroke* | 1.02 (1.0-1.0)  2.11 (1.1-4.1)  1.57 (1.0-2.4)  1.71 (1.1-2.6)  1.68 (1.1-2.5)  2.67(1.1-6.6) | .05  .02  .04  .01  .01  .03 |
| **Patient characteristic** | **above average length of stay in hospital** | |
|  | **OR (95%CI)** | **p-value** |
| PAH*  Digital ulcers*  Private health insurance  Age at onset of SSc^#^  Digital amputation* | 2. 17 (1.2-4.1)  1.52 (0.9-2.3)  0.54 (0.4-0.8)  1.0 (1.0-1.0)  2.22 (1.1-4.6) | .02  .05  .01  .01  .03 |
| **Patient characteristic** | **above average number of ED presentations** | |
|  | **OR (95%CI)** | **p-value** |
| 6MWD, m  Digital ulcers*  Diabetes Mellitus * | 0.99 (0.9-0.9)  1.66 (0.9-3.0)  2.94 (1.1-7.7) | .01  .01  .02 |

Abbreviations: pulmonary arterial hypertension (PAH), gastrointestinal tract (GIT), six minute walk distance (6MWD)

^ median number of hospitalizations 5(2-11), median length of stay (LOS) in hospital 1(1-2.5), median number of ED presentations (n=3)

* manifestations are defined as present if ever present from SSc diagnosis

# age in years at SSc onset defined as age at first SSc clinical manifestation

Supplementary Table 4 Principal diagnosis and procedures for hospitalizations and ED presentations in Victoria (2011-2015)

| **Principal Diagnosis for SSc patients hospitalized between 2011-2015** | | | | |
| --- | --- | --- | --- | --- |
| **Code** | **Frequency** | | | **Description** |
| M349 | 27 (6.3%) | | | SSc |
| D509 | 13(3.0%) | | | Iron deficiency anaemia, unspecified |
| I730 | 10 (2.3%) | | | Raynaud’s syndrome without gangrene |
| R194 | 10(2.3%) | | | Change in bowel habit |
| H269 | 9 (2.1%) | | | Unspecified cataract |
| Z511 | 6 (1.4%) | | | Encounter for antineoplastic chemotherapy and immunotherapy |
| I500 | 6 (1.4%) | | | Congestive heart failure |
| A099 | 5(1.2%) | | | Gastroenteritis and colitis of unspecified origin |
| Z509 | 5(1.2%) | | | Unspecified malignancy neoplasm of skin or other part of trunk |
| L984 | 5(1.2%) | | | Chronic ulcer of the skin |
| **Principle Procedures for SSc patients hospitalised between 2011-2015** | | | | |
| **Code** | **Frequency** | **Description** | | |
| 3047301 | 42 (11.9%) | Panendoscopy to the duodenum with biopsies | | |
| 9619909 | 41 (11.6%) | IV administration of pharmacological agent | | |
| 9555003 | 20 (5.7%) | Allied health intervention, physiotherapy | | |
| 3209000 | 15 (4.2%) | Fibro-optic colonoscopy to cecum | | |
| 3047300 | 12(3.4%) | Panendoscopy to ileum | | |
| 3209300 | 10 (2.8%) | Fibro-optic colonoscopy to cecum | | |
| 1370602 | 9 (2.5%) | Administration of packed cells | | |
| 4270204 | 8 (2.3%) | Cataract procedure | | |
| 3209001 | 7 (1.9%) | Fibro-optic colonoscopy to cecum | | |
| 1220300 | 7 (1.9%) | Polysomnography | | |
| **Principal diagnosis for ED presentations between 2011-2015 in Victoria** | | | | |
| **Code** | **Frequency** | | | **Description** |
| R074 | 17 (5.9%) | | | Chest pain, unspecified |
| R104 | 10 (3.5%) | | | Abdominal pain, unspecified |
| J22 | 10 (3.5%) | | | Acute lower respiratory tract infection, unspecified |
| R060 | 7(2.4%) | | | Dyspnoea |
| J181 | 7 (2.4%) | | | Lobar pneumonia |
| R509 | 6 (2.1%) | | | Drug-induced fever |
| I200 | 5 (1.7%) | | | Unstable angina |
| S628 | 5 (1.7%) | | | Fracture of wrist or hand |
| R11 | 4 (1.4%) | | | Nausea and vomiting |
| N390 | 4 (1.4%) | | | Urinary tract infection |
| **Principal operations for ED presentations between 2011-2015 in Victoria** | | | | |
| **Code** | **Frequency** | | **Description** | |
| 91 | 27 (19.9%) | | Other investigations or procedures | |
| 01 | 22 (16.2%) | | Therapeutic ultrasound of the heart | |
| 99 | 19 (13.9%) | | Transfusion of blood and blood components | |
| 17 | 15 (11.0%) | | Infusion of vasopressor | |
| 51 | 12 (8.8%) | | Implantation of cardiac resynchronisation defibrillator | |
| 41 | 9 (6.6%) | | Operations on bone marrow or spleen | |
| 45 | 6 (4.4%) | | Incision, excision and anastomosis of intestine | |
| 84 | 5 (3.7%) | | Other procedure on MSK system | |
| 11 | 4 (2.9%) | | Magnetic removal of embedded foreign body object in cornea | |
| 21 | 3 (2.2%) | | Operation on the nose | |

Supplementary Table 5 Major diagnostic category of hospitalized patients between 2011-2015

| **Major diagnostic category** | **Frequency** |
| --- | --- |
| Diseases / Disorders of the digestive system | 94 (21.8%) |
| Diseases / Disorders of the MSK System and connective tissue | 92 (21.3%) |
| Diseases / Disorders of the circulatory system | 58 (13.4%) |
| Disease / Disorder of the skin, subcutaneous tissue and breast | 35 (8.1%) |
| Disease / Disorders of the respiratory system | 31 (7.2%) |
| Disease/disorder of the blood and blood forming organs and immunological disorders | 23 (5.3%) |
| Factors influencing health status and other contacts with health services | 19 (4.4%) |
| Disease / Disorder of the nervous system | 14 (3.2%) |
| Disease / Disorder of the eye | 11 (2.6%) |
| Disease / Disorder of the endocrine, nutritional and metabolic system | 11 (2.6%) |
| Disease / Disorder of the kidney and urinary tract | 10 (2.3%) |
| Myeloproliferative diseases / disorders (poorly differentiated neoplasms) | 9 (2.1%) |
| Disease / disorder of the female reproductive system | 9 (1.9%) |
| Disease / Disorder of the ear, nose, mouth and throat | 5 (1.2%) |
| Injuries, poison and toxic effect of drugs | 3 (0.7%) |
| Mental disease and disorders | 3 (0.7%) |
| Disease/ Disorder of the hepatobiliary system and pancreas | 2 (0.5%) |
| Pregnancy, childbirth and puerpium | 2 (0.5%) |
| Infectious and parasitic diseases /disorders (systemic and unspecified sites) | 2 (0.5%) |

Abbreviation: musculoskeletal (MSK).

Supplementary Table 6 Determinants of above median health care cost by univariable logistic regression analysis

| **Patient characteristic** | **Hospital cost** | | **Ambulatory care cost** | | **Medication cost** | |
| --- | --- | --- | --- | --- | --- | --- |
| Patient number | 428 | | 494 | | 531 | |
|  | OR (95%CI) | p-value | OR (95%CI) | p-value | OR (95%CI) | p-value |
| Patient characteristics  Female  Age at SSc onset^#^, year  Age >60 years at SSc onset  SSc disease duration^#^  Tertiary education  Diffuse disease subtype  6MWD, m | 1.01 (0.9-1.0)  1.13 (0.6-1.9)  1.14 (0.7-1.8)  1.01 (0.9-1.0)  0.62 (0.4-0.9)  1.05 (0.7-1.6)  0.9 (0.9-0.9) | .43  .67  .56  .18  .04  .83  < .001 | 1.6 (0.9-2.8)  1.0 (1.0-1.1)  2.0 (1.3-3.2)  1.0 (1.0-1.0)  0.8 (0.5-1.2)  0.7 (0.5-0.9)  0.9 (0.9-0.9) | .10  <.001  .01  .01  .20  .04  .01 | 1.1 (0.7-1.9)  0.9 (0.9-1.0)  0.8 (0.5-1.2)  1.0 (0.9-1.0)  0.9 (0.6-1.3)  2.3 (1.6-3.4)  0.9 (0.9-1.0) | .62  .94  .34  .08  .59  < .001  .05 |
| Clinical manifestations*  Digital Ulcers  Digital amputation  Synovitis  Upper GI involvement  Lower GI involvement  GI involvement  Renal Crisis  ILD  PAH | 1.46 (0.9-2.1)  4.04 (1.8-9.1)  0.87 (0.6-1.4)  1.68 (1.1-2.5)  1.83 (1.2-2.8)  1.78 (1.2-2.7)  2.49 (0.9-7.2)  1.13 (0.8-1.7)  2.27 (1.2-4.2) | .05  < .001  .53  .01  .01  .01  .09  .54  .01 | 1.1 (0.7-1.5)  1.3 (0.7-2.5)  1.9 (1.3-2.9)  2.0 (1.4-2.9)  2.2 (1.5-3.2)  1.9 (1.3-2.7)  1.1 (0.4-2.9)  0.9 (0.7-1.4)  2.8 (1.5-5.3) | .79  .50  < .001  < .001  < .001  < .001  .81  .92  < .001 | 1.6 (1.1-2.3)  1.5 (0.8-2.8)  1.8 (1.2-2.7)  1.6 (1.2-2.3)  2.6 (0.9-6.8)  1.7 (1.2-2.3)  2.4 (0.9-6.4)  2.4 (1.7-3.5)  5.7 (2.9-10.9) | .01  .21  .01  .01  .05  .01  .07  < .001  < .001 |
| Co-morbidities*  IHD  Diabetes Mellitus  Stroke  Current smoker | 2.09 (1.2-3.8)  3.37 (1.6-7.1)  3.79 (1.5-9.6)  0.13 (0.8-1.7) | .02  < .001  .01  .53 | 2.2 (1.3-3.9)  2.6 (1.3-5.2)  1.8 (0.8-3.8)  1.0 (0.7-1.4) | .01  .01  .14  .95 | 1.6 (0.9-2.7)  1.0 (0.5-1.9)  1.9 (0.9-4.2)  1.0 (0.8-1.2) | .08  .99  .07  .90 |
| Instrument scores**  Disease severity score  Highest EUSTAR score | 1.17 (1.1-1.3)  1.25 (1.1-1.4) | < .001  < .001 | 1.18 (1.1-1.3)  1.03 (0.9-1.2) | < .001  .57 | 1.6 (1.5-1.8)  1.3 (1.2-1.5) | < .001  < .001 |

Abbreviations: gastrointestinal (GI), pulmonary arterial hypertension (PAH), interstitial lung disease (ILD), six minute walk distance (6MWD), ischemic heart disease (IHD).

# age in years at SSc onset defined as age at first SSc clinical manifestation, disease duration in years estimated from SSc onset to 2015

* manifestations are defined as present if ever present from SSc diagnosis

** Disease severity is assessed by the physician-rated disease severity assessment score and disease activity is measured by the EUSTAR disease activity score which both range from 0-10, with 0 being no activity / damage and 10 being maximum activity / damage.

^ median hospital cost for the Victorian cohort of ASCS patients (AUD$16,144.50)

Supplementary Table 7 Patient characteristic above and below median ambulatory care and medication cost

| **Patient characteristic** | **Ambulatory care**  **n (%) or mean±SD** | | | **Medication use**  **n (%) or mean±SD** | | |
| --- | --- | --- | --- | --- | --- | --- |
|  | Above median MBS cost  n (%) or mean±SD  median (IQR) | Below median MBS cost  n (%) or mean±SD  median (IQR) | p-value | Above median PBS cost  N (%) or mean±SD  Median (IQR) | Below median PBS cost  N (%) or mean±SD  Median (IQR) | p-value |
| Patient number | 247 (50%) | 247 (50%) | - | 265 (49.9%) | 266 (50.1%) | - |
| Patient characteristics  Age at SSc diagnosis^#^  Female  Caucasian  SSc disease duration^#^  Follow-up duration  Limited disease subtype  6MWD, m (mean)  Autoantibody Profile  Anti-centromere  Anti-Scl70  Anti-RNAP | 49.0±14.1  223 (90.3%)  224 (90.7%)  16.0±10.6  5.9±2.1  187 (75.7%)  368.5±117.1  127 (52.3%)  33 (13.6%)  28 (12.7%) | 43.4±13.6  211 (85.4%)  224 (90.7%)  13.4±9.5  5.5±2.1  167 (67.6%)  412.1±130.4  97 (39.8%)  50 (20.5%)  40 (18.4%) | < .001  .09  .58  .01  .03  .13  < .001  .01  .11  .15 | 46.2±13.8  232 (87.6%)  239 (92.3%)  18.1±10.4  6.4±1.9  168 (63.4%)  372.7±126.1  100 (37.9%)  52 (19.8%)  45 (18.7%) | 46.3±14.5  229 (86.1%)  243 (94.2%)  16.5±10.3  3.4±2.1  211 (79.3%)  398.9±117.3  138 (53.1%)  37 (14.3%)  26 (11.7%) | .96  .62  .37  .04  < .001  < .001  .13  < .001  .10  .04 |
| Clinical manifestations*  Digital ulcers  Digital amputation  Synovitis  PAH  ILD  GIT involvement  SSc Renal Crisis | 123 (49.8%)  21 (8.5%)  83 (33.6%)  38 (15.4%)  79 (31.9%)  167 (67.6%)  9 (3.6%) | 120 (48.6%)  17 (6.9%)  50 (20.2%)  15 (6.1%)  80 (32.4%)  130 (52.6%)  8 (3.2%) | .79  .49  .01  < .001  .92  < .001  .81 | 150 (56.6%)  26 (9.8%)  86 (32.4%)  56 (21.1%)  114 (43.0%)  150 (56.6%)  14 (5.3%) | 119 (44.7%)  18 (6.8%)  56 (21.1%)  12 (4.5%)  63 (23.7%)  117 (43.9%)  6 (2.3%) | .01  .20  .003  < .001  < .001  .01  .06 |
| Co-morbidities*  IHD  Stroke  Diabetes Mellitus | 39 (15.8%)  19 (7.7%)  29 (11.7%) | 19 (7.7%)  11 (4.5%)  12(4.9%) | .01  .28  .01 | 39 (14.7%)  21 (7.9%)  22 (8.3%) | 26 (9.8%)  11 (4.1%)  22 (8.3%) | .08  .06  .99 |
| Instrument scores**  Disease severity score  Highest EUSTAR score | 3.1±1.5  5.0±2.0 | 3.0±1.9  4.3±2.1 | .26  .01 | 5.6±1.9  3.4±1.7 | 3.8±1.9  2.7±1.7 | < .001  < .001 |

Abbreviations: gastrointestinal (GI), pulmonary arterial hypertension (PAH), interstitial lung disease (ILD), six minute walk distance (6MWD), ischemic heart disease (IHD), peripheral vascular disease (PVD), SF-36 physical component score (PCS), SF-36 mental component score (MCS)

# age in years at SSc onset defined as age at first SSc clinical manifestation, disease duration in years from SSc onset to 2015, follow-up in years from recruitment to 2015

* manifestations are defined as present if present ever from SSc diagnosis

** Disease severity is assessed by the physician-rated disease severity assessment score and the activity is by the EUSTAR disease activity score which both range from 0-10, with 0 being no activity / damage and 10 being maximum activity / damage.

^ median ambulatory care cost AUD$11,778.23, median medication cost AUD$2,858.37
